# Whole Exome- and mRNA-Sequencing of an AT/RT Case Reveals Few Somatic Mutations and Several Deregulated Signalling Pathways in the Context of *SMARCB1* Deficiency

**DOI:** 10.1155/2015/862039

**Published:** 2015-08-12

**Authors:** Johanna Sandgren, Stefan Holm, Ana Maria Marino, Jurate Asmundsson, Pernilla Grillner, Monica Nistér, Teresita Díaz de Ståhl

**Affiliations:** ^1^Department of Oncology-Pathology, Karolinska Institutet, 171 76 Stockholm, Sweden; ^2^Department of Women and Child Health, Karolinska Institutet, 171 76 Stockholm, Sweden; ^3^Clinical Pathology/Cytology, Karolinska University Hospital at Solna, 171 76 Stockholm, Sweden

## Abstract

*Background*. AT/RTs are rare aggressive brain tumours, mainly affecting young children. Most cases present with genetic inactivation of *SMARCB1*, a core member of the SWI/SNF chromatin-remodeling complex. We have performed whole exome- and mRNA-sequencing on an early onset AT/RT case for detection of genetic events potentially contributing to the disease. *Results*. A *de novo* germline variant in *SMARCB1*, c.601C>T p.Arg201^∗^, in combination with somatic deletion of the healthy allele is likely the major tumour causing event. Only seven somatic small scale mutations were discovered (hitting *SEPT03, H2BFM, ZIC4, HIST2H2AB, ZIK1, KRTAP6-3*, and *IFNA8*). All were found with subclonal allele frequencies (range 5.7–17%) and none were expressed. However, besides *SMARCB1*, candidate genes affected by predicted damaging germline variants that were expressed were detected (*KDM5C, NUMA1*, and *PCM1*). Analysis of differently expressed genes revealed many dysregulated pathways in the tumour, such as cell cycle, CXCR4 pathway, GPCR-signalling, and neuronal system. *FGFR1, CXCR4*, and *MDK* were upregulated and may represent possible drug targets. *Conclusion*. The loss of *SMARCB1* function leads to AT/RT development and deregulated genes and pathways. Additional predisposing events may however contribute. Studies utilizing NGS technologies in larger cohorts will probably identify recurrent genetic and epigenetic alterations and molecular subgroups with implications for clinical practice and development of targeted therapies.

## 1. Introduction

Atypical teratoid/rhabdoid tumours (AT/RTs) are rare and highly malignant neoplasms of the central nervous system (CNS) usually occurring in early childhood. According to World Health Organization (WHO), AT/RT is classified as a grade IV neoplasm and constitutes 1 of 3 major embryonal tumour entities. The median age for presentation is 20 months with slightly higher frequency in males (M : F, 1.6 : 1) [1]. AT/RT represents approximately 10% of CNS tumours in infants and is most often fatal. Unlike most pediatric brain neoplasms, AT/RTs are in terms of genetic alterations intersimilar and nearly all cases present with deletion and/or mutation of the tumour suppressor gene* SMARCB1* (*INI1*/*hSNF5*) located on chromosome 22 which results in loss of SMARCB1 protein expression [2–5]. This recurrent event seems to be the most common and often the sole genetic aberration reported in AT/RTs [3, 6]. SMARCB1 protein is a core subunit of the ATP-dependent SWI/SNF chromatin remodelling complex that functions as a transcriptional regulator [7]; consequently the loss of its function introduces epigenetic alterations that affect gene expression and might contribute to oncogenesis [8]. An increasing number of mutations detected in epigenetic regulatory genes including additional components of the SWI/SNF complex are also being reported in several other cancer forms, highlighting the importance of alterations in chromatin structure in the development of many malignancies [9].

Due to the aggressiveness of AT/RT tumours, a correct diagnosis is important to ensure the indication of a proper intensive treatment. However, the morphological/histopathological examination of the specimen can as a result of cell heterogeneity be difficult; therefore molecular analysis can have an important role in assisting diagnosis. The determination of* SMARCB1* inactivation with loss of protein expression, mainly assessed by immunohistochemical methods, is now the gold standard procedure applied to confirm diagnosis of suspected AT/RT cases and to differentiate those from morphologically similar tumours.

With the development of next generation sequencing techniques, it is now possible to screen the entire exome/genome for DNA mutations and to profile the complete transcriptome in human biopsies. Such progress has in recent years led to the detection of many novel altered genes and pathways in specific cancers and demonstrated that various molecular subgroups, with differences in prognosis and possibly treatment targets, exist within histologically similar tumours, such as medulloblastomas, glioblastomas, and breast cancer [10–12]. The application of these new methods for the analysis of pediatric brain tumours has then the potential to further increase our knowledge regarding the underlying genetic events involved in the development of these malignancies. Here we report a comprehensive investigation, applying whole exome- and mRNA-sequencing of an early-onset AT/RT tumour diagnosed in a boy during the first year of life.

## 2. Materials and Methods

### 2.1. Patient Material

**Figure 1:**
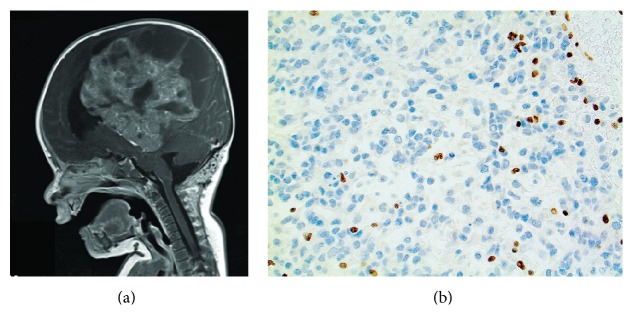
(a) MR scan of patient before operation. (b) Immunohistochemical INI1 staining of the tumour tissue. Staining for INI1 was negative in the tumour cells but positive in endothelial and inflammatory cells.

A three-month-old boy, previously healthy, with no siblings, presented at the local hospital with rapidly growing head circumference, irritability, and inability to raise his head. Clinical examination revealed irritability, bulging fontanel, and head circumference of 44 cm (37 cm at birth) with no neurological deficits. MRI of the head showed a 9 × 8 × 8 cm supratentorial tumour on the left side (Figure 1(a)). The patient was referred to the pediatric intensive care unit and operated on the day after, achieving tumour subtotal resection. Two more operations followed within three weeks for resection of bleeding and residual tumour. MRI of the spine was normal and analysis of the cerebrospinal fluid showed no tumour cells. X-ray of the lungs and ultrasound of the abdomen were both normal.

Fresh tumour tissue and blood samples were collected at the initial surgery at Karolinska University Hospital, Stockholm, Sweden, frozen, and kept at −70°C in a local biobank.

The histopathological examination of the primary specimen revealed a highly cellular neoplasm mainly consisting of rhabdoid cells, relatively rich in eosinophilic cytoplasm and containing globular eosinophilic inclusions. The nuclei were vesicular and peripherally located with prominent nucleoli. Undifferentiated neuroectodermal tumour cells were also observed in some parts. The tumour was highly mitotic, 17/10 HPF; some apoptotic cells and areas of necrosis were also seen. There were foci of inflammatory cells, mainly consisting of lymphocytes. IHC stainings for Vimentin and MAP-2 were positive for most cells. There was patchy positivity for NFP, NSE, CD56, Synaptophysin, GFAP, MNF116, EMA, and EGFR E30. Staining for INI1 was negative in the tumour cells but positive in endothelial and inflammatory cells (Figure 1(b)). Most cells were negative for S-100 and PGR, CD34, Desmin, IDH1, and NeuN. IHC for p21 and p53 showed similar number of stained nuclei, indicating no probable p53 mutation. Ki-67 staining was variable, but ~50% of tumour cells were in proliferative phase. Intraventricular AT/RT, WHO grade IV, was the concluding diagnosis (Table 1).

Intensive chemotherapy treatment (systemic and intraventricular) was started 4 weeks after initial operation according to the EU-RHAB protocol (Prof. Michael Fruhwald, Augsburg). A subdural shunt had to be inserted three months later. Because of local relapse, a reoperation was performed eight months after the first operations and second line chemotherapy was started. The boy stayed on low intensive chemotherapy inclusive intraventricular therapy for one year. During this time, no visible tumour was observed by MRI, but potential malignant cells were detected occasionally in the cerebrospinal fluid. The tumour has though recently relapsed 20 months after diagnosis and the clinical status for the patient includes severe psychomotor developmental delay and feeding difficulties.

This study was conducted with ethical permits approved by local ethical committee.

### 2.2. DNA Extraction, Exome Enrichment, and Next Generations Sequencing

Genomic DNA was extracted from the tumour tissue using phenol-chloroform protocol, including RNAse treatment. Genomic DNA was isolated from peripheral blood using QIAamp DNA Blood Mini Kit (Qiagen, Valencia, California). Enrichment of coding exons was done with Illumina TruSeq Exome Enrichment Kit, 62.1 Mb. The enriched exonic DNA samples were sequenced in a 1/4 lane each in an Illumina Hiseq2000 as 2 × 100 bp paired reads. Exome enrichment, library preparation, and next generation sequencing were performed at* SNP*&*SEQ Technology Platform*, Uppsala, Sweden (http://molmed.medsci.uu.se/SNP+SEQ+Technology+Platform/Sequencing/), according to the manufacturer's instructions (Illumina, San Diego, California).

### 2.3. DNA Sequence Data Analysis

Quality control of the sequencing reads was conducted using FastQC (http://www.bioinformatics.babraham.ac.uk/projects/fastqc/). The fastq sequence reads were aligned to the human reference genome build GRCh37.p5 using CLC Genomics workbench (CLC, Aarhus, Denmark). The alignment settings allowed for successful mapping if at least 70% of reads length had >95% sequence similarity with the reference genome when the mismatch, insertion, and deletion costs were set to 2, 3, and 3, respectively. Reads matching to multiple locations were discarded. Removal of PCR duplicates was performed with Picard (http://picard.sf.net). Further processing of reads including trimming for Illumina adapters, low quality, and short length (below 30 bases) and variant calling were conducted within CLC Genomics workbench. The following criteria were applied for variant calling: (i) a maximum of 2 gaps/mismatches within a 21 bp window, with a minimum base quality of 30, minimum read count of 4, and minimum allele frequency of 5% for tumour and (ii) a maximum of 3 gaps/mismatches in a 21 bp window, with a minimum base quality of 25, minimum read count of 1, and minimum allele frequency of 2% for blood. The variants were annotated according to their overlap with genes and transcripts (UCSC, refSeq GRCh37/hg19 at http://genome.ucsc.edu/) and Sanger cancer census gene (http://cancer.sanger.ac.uk/cancergenome/projects/census/) conservation scores (UCSC), segmental duplications (UCSC), exon number, splice sites, amino acid change, cosmic database v63, The Human Gene Mutation Database (public version, http://www.hgmd.org/), ClinVar (database of mutations and their clinical relevance http://www.ncbi.nlm.nih.gov/clinvar/) and dbSNP v137. Additionally, predictions from SIFT (http://sift.jcvi.org) and Polyphen (http://genetics.bwh.harvard.edu/pph2/bgi.shtml) were added to the list of variants.

Somatic variants were called if they were not present in the blood sample and a minimum read coverage of 8 was achieved (in the blood). Among these, somatic variants presenting with a probably functional effect were selected on the basis of: being previously reported as clinical SNPs (as reported in ClinVar and/or dbSNP v137), present in Cosmic database, determined to be probably damaging/damaging according to Polyphen/SIFT, overlapping a splice sites, resulting in a stop codon or were indels giving rise to amino acid change. The variations were further filtered out if detected in any of 97 pieces of control exomes data obtained from noncancer patients (including samples from the 1000 genome project) or were listed in dbSNP v137, with a population frequency higher than 1%. Moreover, mutations located within segmental duplications or in the* TTN*,* MUC*, and* OR* genes were removed based on the known risk of introducing false positives due to high sequence similarities and associated alignment problem. The resulting list of somatic mutations was visually inspected across the read alignments. Germline variants were processed in a similar way with the exception that the variants were required to be present in both tumour and blood samples with a minimum variant frequency of 20% and a minimum read count of 4 to be considered.

### 2.4. RNA Extraction and Next Generation Sequencing

Total RNA was extracted from the tumour tissue according to Trizol protocol followed by Qiagen RNeasy Mini cleanup kit and DNase treatment. RIN values were checked on a Bioanalyser (Agilent) and polyA-tailed mRNA isolation according to Illumina library preparation instructions was conducted. The enriched mRNA was sequenced in a 1/8 lane of an Illumina Hiseq as 2 × 100 bp paired reads at the Karolinska Institutet Science for Life Laboratory.

We used two public control data sets, SRS151250 and SRS173568, that represent mRNA-seq data from equally pooled amounts of total RNA from the frontal part of the superior frontal gyrus of postmortem brains at different age points. The age in SRS151250 was from 1 to 35 days with 3 males and 2 females and the age in SRS173568 was from 182 to 274 days with 3 males and 2 females. Raw data generated and used here were 76 bp paired reads sequenced on Illumina GAII (http://trace.ddbj.nig.ac.jp/index_e.html).

### 2.5. mRNA Sequence Data Analysis

The sequence reads in fastq format were quality checked using FastQC (http://www.bioinformatics.babraham.ac.uk/projects/fastqc/) and aligned to the human reference genome build GRCh37/hg19 using Tophat v1.4 with the following settings: tophat -p 8 -r 70 -a 8 -m 0 -i 70 -I 500000 -g 20 –library-type fr-unstranded –max-insertion-length 3 –max-deletion-length 3 –coverage-search –min-coverage-intron 50 –max-coverage-intron 20000 [13]. Subsequently duplicate removal was performed using Picard. Variant calling on the tumour RNA data was conducted in CLC Genomics workbench applying the same settings as for the tumour DNA sample. FPKM values were calculated with Cufflinks v2.1.0 using Ensembl annotations for genes and transcripts. To identify differentially expressed genes and transcripts between the tumour sample and the control sets we used Cuffmerge to combine the Cufflinks assemblies and then Cuffdiff v2.1.0 to find significant changes. Cuffdiff settings were as follows: –library-norm-method geometric –dispersion-method pooled -p 8 -c 5 –FDR 0.050000 [14]. The online version of Galaxy (https://usegalaxy.org/) was used to run FastQC, Tophat, Picard, Cufflinks, Cuffmerge, and Cuffdiff [15]. Downstream analysis and visualization of Cuffdiff output were conducted in R with the package CummeRbund [16].

### 2.6. Gene Ontology and Pathway Analysis

Gene Ontology (GO) and gene set overlap calculations were performed on the significantly upregulated and downregulated genes separately for determination of overrepresentation of specific biological functions, including cell signaling pathways. Gene sets used include curated collections from pubmed and online databases: canonical pathways, KEGG, BIOCARTA, and REACTOME (http://www.broadinstitute.org/gsea/msigdb/annotate.jsp) [17]. GO analysis for overrepresented biological processes was performed using DAVID (http://david.abcc.ncifcrf.gov/home.jsp) [18].

## 3. Results

The AT/RT patient investigated in this study presented with symptoms already at the age of three months (see case description in Section 2). Imaging studies revealed a large supratentorial tumour (Figure 1(a)) and the histopathological examination confirmed an AT/RT diagnosis including negative INI1 staining (Figure 1(b)). Despite surgical treatment and intense chemotherapy, local relapse occurred after eight months. Additional chemotherapy treatment was then started. Potential malignant cells were however found in cerebrospinal fluid and the boy has been reoperated for a second relapse 20 months after initial diagnosis (Table 1).

### 3.1. Few Somatic Mutations with Low Allele Frequencies Not Detected in RNA

To discover tumour specific mutations, we performed whole exome-sequencing on DNA derived from the patient's tumour and blood, which generated 4.9 × 10^7^ and 8.6 × 10^7^ paired reads for each sample. The average read coverage for the targeted region (62.1 Mb) after trimming of adapters, alignment, and duplicate read removal was 19x and 49x for tumour and blood, respectively (Supplementary Table 1A  in Supplementary Material available online at http://dx.doi.org/10.1155/2015/862039). To identify somatic mutations, we compared the variants present in blood and tumour samples and selected those found only in the tumour, with sufficient read coverage in blood; see Section 2. After filtering out common and nondamaging variants (see Section 2), seven somatic mutations remained. This indicated a mutation rate of as low as 0.145 mutations per Mb (Tables 1 and 2). We further inspected the allele frequencies of these mutations and could observe that all these variants were found at a subclonal frequency levels (range 5.7–17%), indicating that they were present only in a fraction of tumour cells and consequently they probably represent passenger or later events that occur after tumour initiation.

We also performed in depth transcriptome analysis. For that, isolated polyA enriched mRNA from the tumour sample was paired end sequenced, producing 2.1 × 10^7^ aligned reads after duplicates removal (Supplementary Table 1B). The mRNA seq data allowed us to investigate the variant specific expression of transcribed genes. Interestingly, none of the seven somatic mutations determined by exome-sequencing (though present at low DNA allele frequency) were found in tumour RNA (Table 2).

### 3.2. Germline Candidate Variants Including the Mutation and Somatic Loss of* SMARCB1*

**Figure 2:**
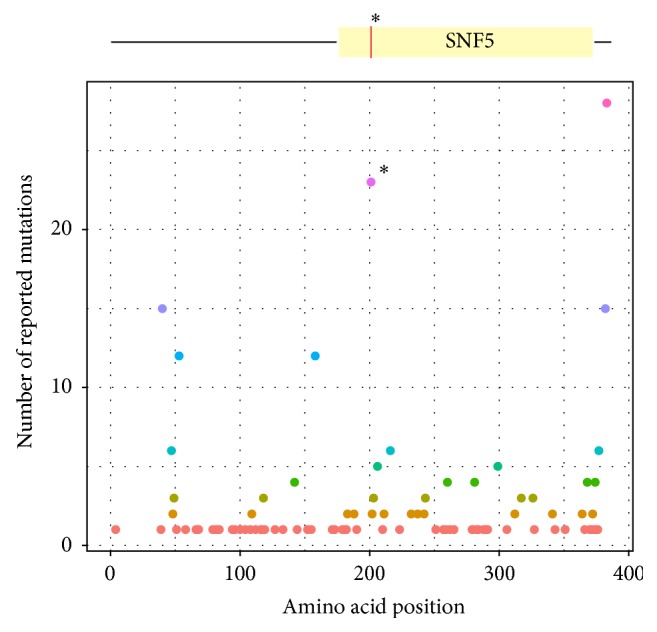
SMARCB1 protein and reported mutations. Schematic view of SMARCB1 and the distribution of reported mutations along the protein. The germline mutation detected (red line) is located in the SNF5 domain. This alteration is the second most common mutated variation reported in Cosmic database as illustrated in the plot (*∗* denotes the position for the detected mutation). Depicted are all reported substitutions and insertions/deletions mutations with a maximum size of 3 nucleotides. The majority of cases are AT/RTs. The different colors in the plot indicate the different frequencies of reported mutations.

Furthermore, we investigated the presence of possible germline disease causing mutations. After applying appropriate filtering steps (see Section 2), 205 uncommon and predicted damaging germline variations affecting 200 genes were found (Table 1 and Supplementary Table 2). Among these, the most likely tumour causing event in this patient seems to be a germline nonsense mutation in* SMARCB1* gene, c.601C>T p.Arg201^*∗*^. This C>T substitution which results in a truncated SMARCB1 protein is not reported in the general human population (dbSNP v137) but is the second most common* SMARCB1* mutation reported in the catalogue of somatic mutations in cancer, Cosmic database (June 2013) (Figure 2). Moreover, the allele frequency observed for the germline mutation (c.601C>T p.Arg201^*∗*^) raised from 57.8 in blood to 87.5 in the tumour, indicating a somatic loss of the healthy allele (however not reaching 100% frequency probably due to the existence of some normal cells in the tumour sample). In addition, we confirmed the absence of SMARCB1 protein expression by IHC staining (Figure 1(b)). Consequently, the presence of a germline mutation and the acquired somatic loss of the healthy allele lead to 2 hits inactivation and complete deficiency of SMARCB1 function.

Among the remaining genes affected by uncommon germline variations predicted to be deleterious for the protein, eight additional candidates were selected based on the facts that the mutations were reported in Cosmic database and/or the genes were included in the Sanger cancer Gene Census list, which contains genes that have been causally implicated in cancer (Table 3). Of those eight candidates,* ARID1A*,* CNTN6*, and* CSTA* presented with the same mutations already annotated in Cosmic. However, none of these variants have previously been reported in CNS tumours and all three genes have been found to harbour mutations in very low fractions (0.15–0.8%) of the catalogued CNS tumours, none of which were AT/RTs. The other five genes:* RECQL4*,* MYCL1*,* KDM5C*,* NUMA1*, and* PCM1* are documented in the cancer Gene Census list. In our case,* KDM5C* carries a novel predicted damaging germline mutation. This variant may be a potentially interesting finding as inactivating mutations in this gene that encode a histone demethylase most likely affect the epigenetic state which in turn could have implications in AT/RT tumorigenesis.

**Figure 3:**
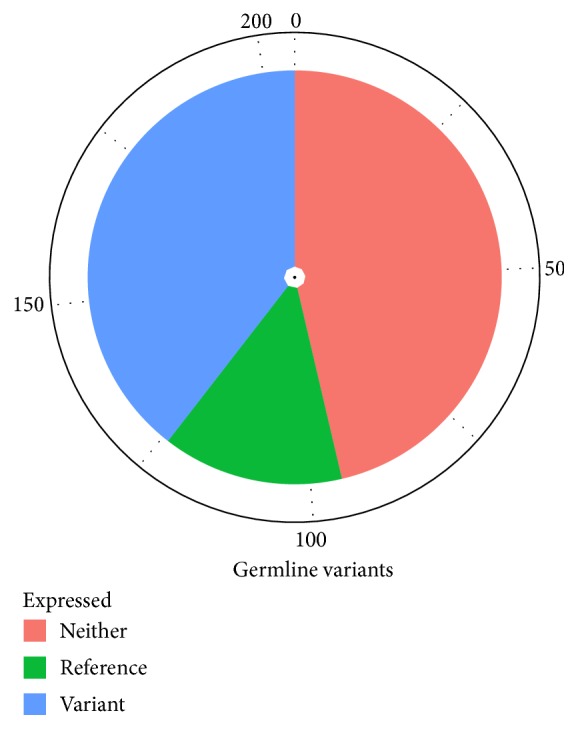
Fraction of expressed/not expressed uncommon and damaging germline variants.

We used the mRNA seq data to investigate the expression of these potentially important variants in the tumor. Interestingly, several of these germline variants were found to be expressed in the tumour, including the nonsense mutation in* SMARCB1* (Table 3). The allele frequency of the* SMARCB1* mutation in mRNA seq data was 75%, also supporting the loss of the wild type allele and the consequently biallelic inactivation of* SMARCB1* in the tumour (but as in exome data not reaching a frequency of 100% due to some nontumor cells contamination, Supplementary Table 2). Two transcripts are annotated in CCDS for* SMARCB1*, NM_003073.3 with protein ID NP_003064.2 and NM_001007468.1 with protein ID NP_001007469.1, where the latter gives rise to a protein nine amino acids shorter. Both these transcripts seem to be expressed in the tumour based on the FPKM values from Cuffdiff transcripts analysis (Supplementary Figure 1) and by visual inspection of aligned reads in exon 2, where these two transcripts differ at genomic position 24134055–24134081 bp. The shorter transcript lacks this segment and consequently some reads do not align to this region and instead continue to exon 3 (Supplementary Figure 2). Concerning* KDM5C* transcription, the detected germline variant was expressed with an allele frequency of 100%, which is the expected value as the patient investigated here is a boy and the gene is lying on chromosome X. The germline variants observed in the cancer related genes* NUMA1* and* PCM1* were also transcribed and detected in the RNA data (Table 3). These genes have functions related to mitotic spindle establishment and centrosome assembly, respectively. In total 81 of the 205 uncommon and predicted damaging germline variants were found in the RNA data; for additional 29, only the common allele was detected and for the remaining neither allele was expressed (Figure 3 and Supplementary Table 2).

### 3.3. Several Upregulated and Downregulated Genes and Pathways with Implications in Cancer

Analysis of differently expressed (DE) genes in the AT/RT compared to healthy, age-matched control samples resulted in 3813 significantly DE genes. Of those, 1661 were upregulated and 2152 were downregulated (Supplementary Table 3). Of the 200 genes carrying uncommon and damaging germline variants, 31 were significantly DE in the tumour and 12 of them expressed the variant (Supplementary Table 2). Among the nine selected cancer associated genes with germline variants (Table 3)* SMARCB1* was downregulated (Supplementary Figure 1) and so was* CNTN6,* whereas* MYCL* was upregulated (Supplementary Table 3).

**Figure 4:**
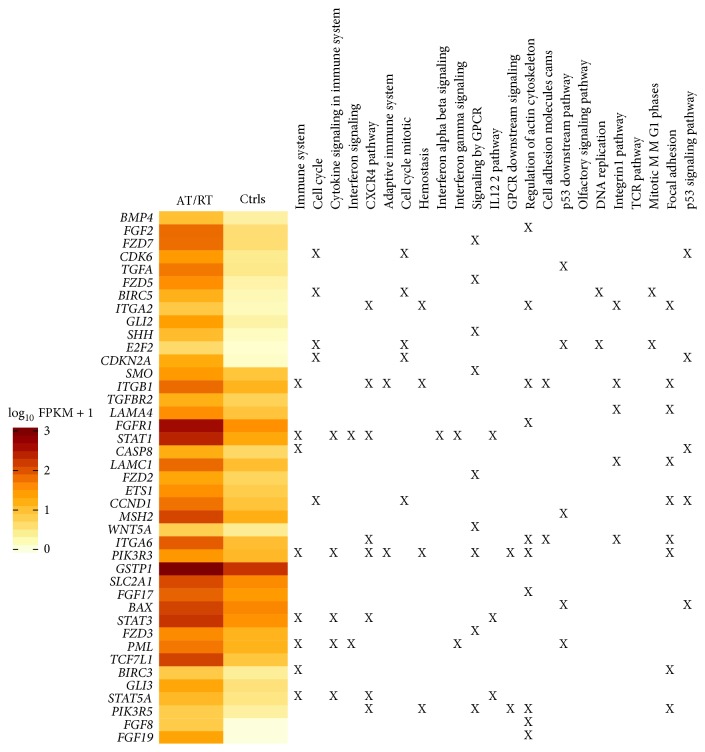
Heat map of all significantly upregulated genes in gene set pathways of cancer for AT/RT sample versus controls. Also indicated with “X” if a gene is included in any of the additional 24 most significantly enriched gene sets, for all the upregulated genes.

Statistical gene set overlap evaluation using the comprehensive Molecular Signature data base (MSigDB) on all upregulated genes in the tumour revealed that immune system, cell cycle, CXCR4 pathway, pathways in cancer, GPCR signaling, regulation of actin cytoskeleton, and p53 downstream pathway were among the top 20 significantly activated gene sets (Supplementary Table 4). A heat map with all upregulated genes involved in pathways of cancer is shown in Figure 4. Many of these genes, for example,* PIK3R3, PIK3R5, ITGB1, ITGA2, ITGA6, STAT1, STAT5A*, and* STAT3*, are also involved in the above mentioned CXCR4 pathway and some of them also contribute to the regulation of actin cytoskeleton and GPCR signaling, for example,* CXCR4, RHOC,* and* RGS1* (Figure 4).

*FGFR1* was another upregulated gene acting in pathways of cancer and also playing a role in the actin cytoskeleton signaling. Interestingly, ligands for this receptor,* FGF2, FGF17, FGF8*, and* FGF19*, were also overexpressed in the tumour. Moreover, genes in the WNT, SHH, and BMP pathways such as* WNT5A, FZD7*, and* FZD5, SHH, SMO*, and* GLI2* as well as* BMP4* were likewise among the upregulated genes and were included in pathway of cancer gene set (Figure 4). Overexpression of* BMP4* and several members of the BMP pathway have previously been correlated to bad prognosis in AT/RT [19]. The search among all the DE genes, for the 87 known BMP members, according to Birks et al. [19], revealed that 25 additional BMP genes were significantly dysregulated in the tumour; of those, 14 were among the upregulated ones and 11 were among the downregulated ones (Supplementary Table 3). Gene ontology analysis carried out with all the upregulated genes showed an enrichment of partly similar biological processes: mitosis, chromosome segregation, immune system development, embryonic development, and cell proliferation among others (Supplementary Table 6).

Among the top overexpressed genes,* MDK*,* S100A4*, and* HMGA2* are worth mentioning.* MDK,* which ranked 25th amongst all 1661 upregulated genes (log2 FC: 4.3 and FPKM: 706.7), acts within the activated integrin pathway. MDK is known to promote proliferation and migration and to inhibit apoptosis. Moreover, it has been correlated to bad prognosis in glioblastoma [20].* S100A4* and* HMGA2* are also interesting as both have been implicated in cancer. They ranked 3rd (log2 FC: 8.5 and FPKM: 874.3) and 6th (log2 FC: 8.1) among most upregulated genes, respectively.

MSigDB gene set overlap calculations performed with the significantly downregulated genes revealed that signaling by GPCR, developmental biology, axon guidance, neuronal system, and ion channel transport were among the top 20 overrepresented pathways (Supplementary Table 5). Very similar biological processes were observed as a result of gene ontology analysis (Supplementary Table 7).* SMARCB1* was as mentioned above downregulated in the tumour and so were* DOCK4, SPOCK1, PTN*, and* ATP1B1* which are known to have expression levels that correlate with* SMARCB1* [21–23]. These genes play roles in neuronal system development and have previously been shown to be downregulated in rhabdoid tumours [22, 23]. Neuronal developmental markers* SOX11* and* SNAI2* had also significantly lower expression compared to controls as well as the* GNAI1* gene. Another potential interesting gene is* MEIS2* which was the second most underexpressed gene.* MEIS2* is involved in transcriptional regulation and it has also been shown to have decreased expression in poor prognosis prostate cancer [24].

## 4. Discussion

AT/RT is primarily a disease of children under three years of age [1] and represents approximately 1.3% of paediatric brain tumours and 20% of all embryonal tumours [25, 26]. Treatment approaches include resection, high dose chemotherapy, and radiation, the latter being used if the patient is over 18 months of age. The patient studied here was operated on for both primary tumour and relapses and suffered from the disease and the intense treatment received.

Based on our findings, the disease causing mutation is a* de novo* germline variant in* SMARCB1*, c.601C>T p.Arg201^*∗*^ occurring in the patient, that when combined with the somatic loss of the healthy allele leads to the complete loss of SMARCB1 function in the tumour cells. The germline mutation resides in the conserved SNF5 domain of the protein, which is a relatively common hotspot for alterations. There are more than 20 cases reported in Cosmic database with the same nonsense mutation and the majority of them are AT/RTs. The mutation in the patient was also confirmed by Sanger sequencing (clinical analysis, data not shown). Moreover, sequence analysis of parents' blood DNA sequencing (clinical analysis, data not shown) could not identify the presence of this* SMARCB1* mutation. This supports a* de novo* origin of the constitutional mutation in the child and excludes the parents as carriers, with the exception of the very unusual presence of gonadal mosaicism. Germline alterations in* SMARCB1* give rise to the Rhabdoid Tumour Predisposition Syndrome (RTPS) which is manifested by development of malignant rhabdoid tumours in infancy and early childhood [27, 28]. This also explains the early disease onset, with the patient being only three months old. Due to the relatively high frequency (up to 35%) of germline mutations in* SMARCB1* in AT/RT and the incomplete penetrance of inherited mutations in some cases [29], the screening of constitutional mutations in* SMARCB1* in patients diagnosed with AT/RT is now a routine procedure at Karolinska University Hospital. Results from such testing can bring out important information used in genetic counselling for families with an affected child and importantly discovery of a* SMARCB1* mutation also confirms diagnosis.

In the present case, loss of the healthy* SMARCB1* allele in the tumour led to biallelic inactivation of the gene that is reflected by the increased allele frequency of the germline mutation in the tumour compared to blood and by the negative IHC staining for SMARCB1 protein. Another striking observation was the limited number of somatic variants detected that were predicted to be damaging. Only seven, and all of them found at subclonal frequency level were the highest allele frequency was 17%. Moreover, none of them were expressed. Furthermore, none of these genes are known as cancer drivers neither have the identified mutations been reported in Cosmic or in other AT/RT tumours [3]. Therefore, these somatic changes are not likely the ones driving tumour development. Similar trends have been previously reported by Lee et al. [3] who investigated 32 AT/RT cases for somatic changes and found only 172 mutations; many of them were detected at low allele frequency resulting in an overall very low mutation incidence compared to that in the present case (0.19 versus 0.14 mutations per Mb, resp.). The number of somatic mutations in AT/RT then appears to be even lower than that found in other malignant paediatric brain tumours such as medulloblastomas and much lower than that in adult malignant brain tumours as anaplastic astrocytomas and glioblastomas [10, 30, 31]. It might be that in the case of AT/RT the biallelic inactivation of* SMARCB1*, despite being the main recurrent somatic aberration, is also a sufficient event to promote tumour development. This is further supported by a study where 115 selected cancer associated genes were sequenced in 25 AT/RT samples. All cases presented with* SMARCB1* alterations, apart from one, where a mutation in* NRAS* was found instead [32]. DNA copy number profiling has also shown that often there is only a single gross genomic aberration present in almost all tumours, which is loss of chromosome 22, where* SMARCB1* is located [6].

We also searched for additional germline mutated genes other than* SMARCB1*, which could contribute to tumour predisposition. Experiments carried out in conditional* SMARCB1* knock outs in mouse fibroblast cells have shown that the silencing of* SMARCB1* results in growth arrest and p53-mediated cell death transformation [33]. This might suggest that additional genomic events or the specific cellular environments in determined cell types are required for SMARCB1 associated malignant transformation. To note, there are also many studies showing that reintroduction of wild type SMARCB1 in human AT/RT cells leads to cell cycle arrest and its tumour suppressor function is firmly established [34–37]. It is though interesting that we found a few possibly damaging variants in cancer associated genes in the patient's constitutional DNA.* KDM5C*, for example, is an interesting gene with epigenetic implications. It is situated on chromosome X and subsequently only the uncommon allele is expressed in this male patient.* KDM5C* encodes a histone demethylase specific for histone 3 lysine 4 that functions as a transcriptional repressor. This gene has been reported to be mutated in cancer including medulloblastoma (Cosmic).* NUMA1* and* PCM1* are additional genes for which the predicted damaging germline variants were expressed in the tumour. Both* NUMA1* and* PCM1* codes for proteins with important roles in cell division, so alterations in these proteins may have an impact on the normal cell cycle. It is important to document the presence of these variants; however, the possible synergistic effect of any additional genetic event(s) beside* SMARCB1* aberrations responsible for AT/RT development is difficult to evaluate from a mutational screen of a single case.

Transcriptome analysis revealed several deregulated genes and pathways in the AT/RT compared to control samples. In accordance with a highly proliferative tumour, cell cycle was among the top significant enriched processes for upregulated genes. Additionally, p53 pathway and pathways in cancer, the latter including several SHH pathway members (*SHH*,* SMO*, and* GLI2*), seemed to be activated. Interestingly, and in agreement with our results, cell cycle and p53 and SHH pathways were shown to be enriched among upregulated genes in an expression microarray study performed on 20 AT/RT and 10 kidney rhabdoid tumours (KRT) [23]. Moreover, aberrant activation of SHH pathway has also been observed in SMARCB1-deficient cells [38]. WNT pathway members (such as* WNT5A*,* FZD7*, and* FZD5*) were also upregulated in the AT/RT case studied here, which is of interest as WNT pathway has been associated with a cluster of AT/RTs with significantly shorter survival [19]. Both WNT and SHH are important developmental pathways known to have disturbed activation in other paediatric malignancies [10]. Furthermore, the upregulation of* BMP4* in the tumour, which is a signaling cytokine growth factor, acting within the here enriched pathways of cancer, is also a noteworthy finding since* BMP4* has been reported to be the top DE gene between clusters of AT/RT tumours with different survival outcome, showing a clear overexpression in tumours with short survival [19]. The BMP pathway is a crucial signaling cascade acting during embryo growth, particularly important for CNS development, but it is also essential in maintaining homeostasis in adult tissues [39, 40].* BMP4* overexpression has also been shown to promote invasion in different cancer cells [41–43]. Therefore, the upregulation of* BMP4* in this case with several tumour relapses might be supportive of its role as a marker for bad prognosis.

We also found a strong upregulation of the cancer associated gene* FGFR1* and several FGF ligands, all of them acting within the pathways of cancer and regulation of actin cytoskeleton. Of note,* FGFR1* was overexpressed in AT/RT in the recent expression array study mentioned above [23]. The gene is also known to be amplified in squamous cell lung and breast cancer (so is* FGF2*) and to contain activating mutations in low grade astrocytoma [44–47]. Signaling via FGFRs activates signaling transduction pathways including Ras-MAPK, PI3-AKT, and PLC*γ*-PKC, mediating cell survival, proliferation, differentiation, migration, and drug resistance. Importantly, these pathways could be modulated in AT/RT as several FGFRs-targeted therapies have been developed which have been shown to reduce proliferation of cancer cells [48]. Another upregulated pathway which is worth mentioning is CXCR4. CXCR4 mediates cascades such as PI3K/AKT, JAK/STAT, and Rac/Rho, which are involved in cancer and promote tumour progression. Various cancers including glioma, neuroblastoma, and leukemia show overexpression of CXCR4 and since their blockade may have implications for therapy several inhibitors have been developed and are, at least for hematopoietic cancers, being tested in clinical trials [49, 50]. Not much attention has been given to this pathway in AT/RT, even though the downstream signaling effectors such as AKT and Rho have been seen to be upregulated [21, 51]. CXCR4 may be therefore a promising candidate for drug targeting in AT/RT cases which show such aberrant signaling activation.

Specific additional upregulated genes in the AT/RT case that might be of importance and also possible candidates for biomarkers and/or drug targeting are* MDK*,* S100A4*, and* HMGA2*. MDK is a secreted growth factor known to promote proliferation, migration, antiapoptosis, transformation, and angiogenesis. Interestingly, previous reports have also shown high expression of* MDK* in advanced tumours which correlated to serum levels and tumour progression [20, 52]. MDK could also be considered as target for therapy as its inhibition has shown to potentiate the effect of cytotoxic drugs [52]. S100A4 plays important roles in several cellular processes, including the regulation of cell cycle and differentiation.* S100A4* is upregulated in many types of cancer in which it has been clearly linked to tumour progression and bad prognosis [53–58]. HMGA2 functions as a transcriptional regulator and as a component of the enhanceosome [59]. HMGA2 is expressed during early development but also in late stage or invasive cancer forms [60, 61]. Both* S100A4* and* HMGA2* were found to be overexpressed in AT/RT [23].

Regarding downregulated transcripts in AT/RT, many genes involved in developmental biology, axon guidance, and neuronal system were found. Decreased expression of genes associated with neuronal development was seen in RT with SMARCB1 loss in comparison with other kidney tumours [22] and the same above mentioned processes were found to be downregulated in short survivor AT/RT patients [19], which indicates aberrant neuronal functions.* DOCK4* and* PTN*, for example, were significantly downregulated genes in this AT/RT case. Both genes were recently shown to be underexpressed in AT/RT and/or RT [22, 23] and to present with concordant differential downregulation with* SMARCB1* [21].* GNAI1* is moreover included in the downregulated gene sets of GPCR, axon guidance, and neuronal development in our analysis. It regulates cell proliferation and differentiation [62, 63] as well as cancer migration and invasion [64].* GNAI1* was also previously described as underexpressed in AT/RTs [23].

However, several early neural cell genes that were downregulated in KRT [22] were not seen to be significantly downregulated in our case. Similar results were observed in a recent gene expression array study of AT/RTs [23]. Specific genes seen to be dysregulated previously, such as the histone methyl transferase* EZH2* that is a part of PRC2, were not upregulated in our study either. So, despite often only carrying a seemingly simple genetic alteration, leading to the inactivation of* SMARCB1,* AT/RT tumours are heterogeneous when it comes to the transcripts/pathways affected and this may be reflected in patient outcome. Predisposing genetic events, environmental factors, and/or different cell of origin may represent some features that influence this heterogeneity.

## 5. Conclusions

In summary, regardless of these gene expression differences observed between studies and samples, striking common abnormalities exist within AT/RTs, and several pathways and genes appear recurrently dysregulated. Among these cell cycles, p53 pathway and SHH as well as the specific genes mentioned above,* FGFR1*,* S100A4*, and* HMGA2*, were repeatedly upregulated, whereas neuron differentiation and neuronal development pathways as well as genes with neuronal functions,* DOCK4*,* PTN*, and* GNAI1*, were reiteratively downregulated. Consequently they should be taken into account as potential biomarkers and/or targets for therapy. The recurrence of these findings across studies is, beside its biological importance, a validation of our analysis. AT/RT is a relative unusual tumour and there is currently a lack of comprehensive genomic and epigenomic studies including larger set of patients. However, with the development and more extended application of next generation sequencing techniques as well as improvements in the collection of appropriate material in bio banks, more reports will likely be seen. Such efforts should reveal if there are more frequent genetic aberrations to be found that could define molecular subgroups with clinical implications. The increased knowledge will aid in the development of targeted therapies for improved quality of life and hopefully overall survival for these unfortunate children.

## Supplementary Material

Supl. Table 1A. Read mapping and coverage data. Exome-seq Data.
Supl. Table 1B. Read mapping mRNA-seq data.
Supl. Table 2. Germline variants with RNA variants and DE genes indicated.
Supl. Table 3. Cuffdiff results, significantly DE genes with BMP-genes indicated.
Supl. Table 4. Gene set overlap for up regulated genes.
Supl. Table 5. Gene set overlap for down regulated genes.
Supl. Table 6. GO for up regulated genes.
Supl. Table 7. GO for down regulated genes.
Supl. Fig. 1. FPKM values for the two CCDS *SMARCB1* transcripts for AT/RT and Controls. NCBI accession nomenclature is used.
Supl. Fig. 2. RNA reads alignment at exon 2 of *SMARCB1*. Genomic region Chr. 22: 24134055- 24134081 is indicated between the two red vertical lines. The yellow region on the top, represents exon 2, with introns on both sides, shown in blue. Aligned paired reads are shown in blue, while single reads are shown in red or green. Dotted lines connect each end of a read, that have been mapped across exon-exon boundaries.

## Figures and Tables

**Table 1:** Clinical data and mutation summary.

Sex	Male
Age at diagnosis (months)	3
Localization	Left supratentorial
Operation^a^	Subt. resection + residual
IHC^b^	INI1 negative
Therapy^c^	EU-RHAB protocol
Recurrence (months)	8 and 20
Survival (months)	23
Number of somatic mutations	7
Somatic mutation rate per Mb	0.145
Transitions/Transversions	2/3
Deletions/Insertions	1/1
Number of postfiltering germline variants^d^	205

^a^Initial operation performed. Subt. = Subtotal. Residual = additional

operation had to be done to remove residual tumour.

^b^Selected IHC marker from pathology analysis.

^c^EU-RHAB includes several chemotherapy drugs including Doxorubicin, Ifosfamide, Carboplatinum, Etoposide, Vincristine, and Cyclophosphamide.

^d^The uncommon and predicted damaging variants.

**Table 2:** Somatic point mutations.

Reference genome coordinates	Gene	Predicted protein alteration	Mutation allele frequency	Mutation expressed	Reference allele expressed (base coverage)
chr22: 42390734delG	*SEPT03 *	NP_061979.3:p.Glu343fs	13.8	no	Yes (35)
chrX: 103294635C>T	*H2BFM *	NP_001157888.1:p.Thr31Met	6.7	no	No (0)
chr3: 147113718insA	*ZIC4 *	NP_115529.2:p.Phe204fs NP_001161850.1:p.Phe254fs NP_001161851.1:p.Phe242fs	7.4	no	Yes (55)
chr1: 149859313G>T	*HIST2H2AB *	NP_778235.1:p.Leu52Met	9	no	No (0)
chr19: 58102066A>T	*ZIK1 *	NP_001010879.2:p.Glu296Val	14.6	no	Yes (11)
chr21: 31965069A>C	*KRTAP6-3 *	NP_853636.3:p.Tyr102Ser	17	no	No (0)
chr9: 21409401G>A	*IFNA8 *	NP_002161.2:p.Ala76Thr	5.7	no	No (0)

NCBI accession nomenclature is used for proteins.

**Table 3:** Candidate germline variants.

Reference genome coordinates	Gene	Predicted protein alteration	Variant allele frequency	Mutation expressed	Reference allele expressed
chr22: 24145582C>T	*SMARCB1 *	NP_001007469.1:p.Arg192^*∗*^ NP_003064.2:p.Arg201^*∗*^	87.5	Yes	Yes
chr8: 145739905G>A	*RECQL4 *	NP_004251.3:p.Ser542Phe	55.3	No	Yes
chr1: 27100206insGCA	*ARID1A *	NP_006006.3:p.Gln1334insGln NP_624361.1:p.Gln1334insGln	28.2	No	Yes
chr1: 40366547C>G	*MYCL1 *	NP_005367.2:p.Cys217Ser	55	No	No
chrX: 53227814C>G	*KDM5C *	NP_001140174.1:p.Glu725Gln; NP_004178.2:p.[Glu792Gln]	100	Yes	No
chr11: 71727189G>A	*NUMA1 *	NP_006176.2:p.Arg454Trp	80	Yes	Yes
chr8: 17815114G>A	*PCM1 *	NP_006188.3:p.Glu624Lys	45.5	Yes	Yes
Chr3: 1269653C>T	*CNTN6 *	NP_055276.1:p.Arg112Trp	80	No	No
chr20: 44520260delTG	*CTSA *	NP_001161066.1:p.Leu36fs NP_000299.2:p.Leu36fs NP_001121167.1:p.Leu18fs	26.7	No	Yes
