# Supplementary figures and images for: Whole Exome- and mRNA-Sequencing of an AT/RT Case Reveals Few Somatic Mutations and Several Deregulated Signalling Pathways in the Context of SMARCB1 Deficiency
Source: Biomed Res Int. 2015 Aug 12;2015:862039. doi: 10.1155/2015/862039 (PMC4780067; doi:10.1155/2015/862039)

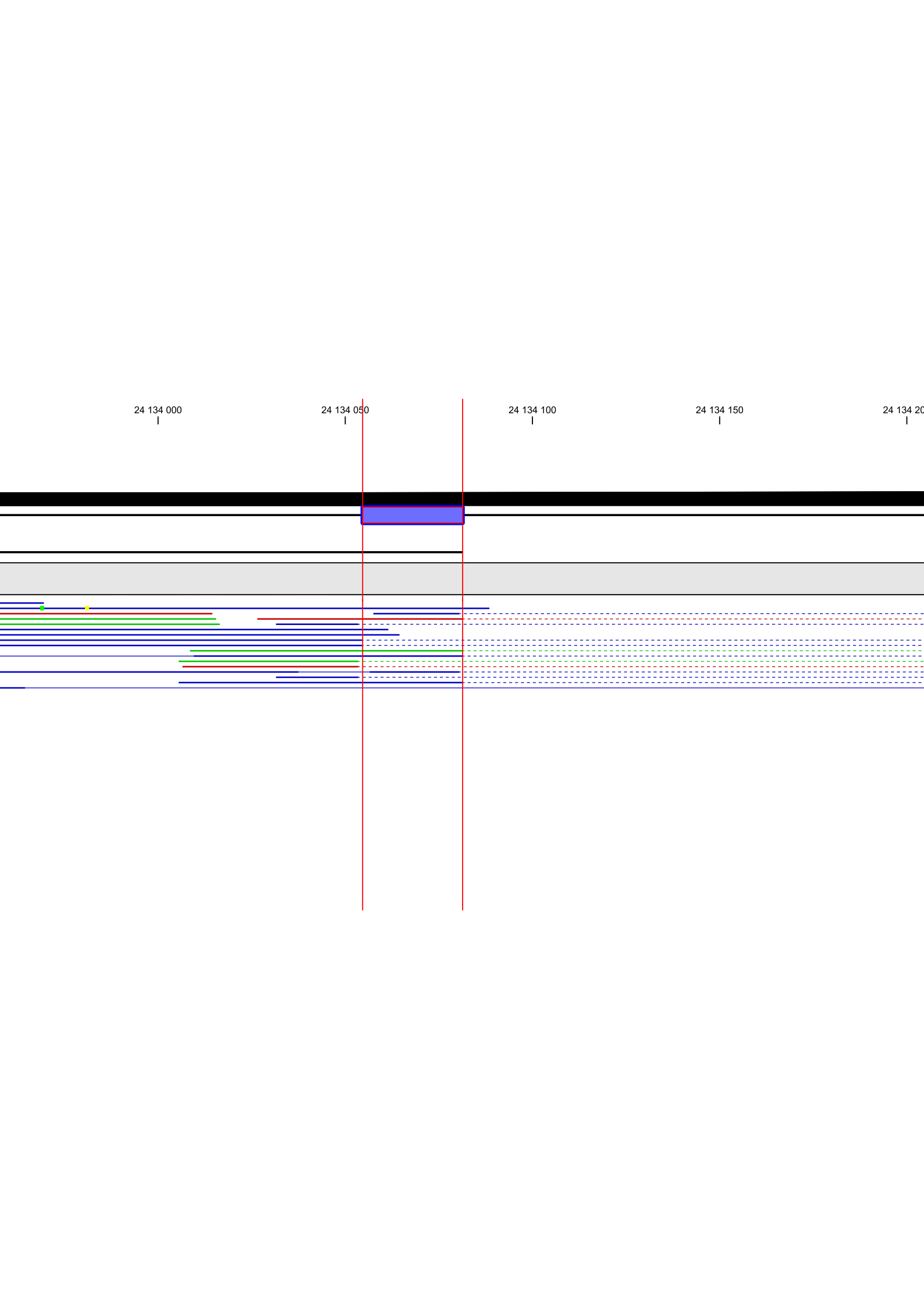

Supplement: Supplementary file 1 — Supl. Table 1A. Read mapping and coverage data. Exome-seq Data. Supl. Table 1B. Read mapping mRNA-seq data. Supl. Table 2. Germline variants with RNA variants and DE genes indicated. Supl. Table 3. Cuffdiff results, significantly DE genes with BMP-genes indicated. Supl. Table 4. Gene set overlap for up regulated genes. Supl. Table 5. Gene set overlap for down regulated genes. Supl. Table 6. GO for up regulated genes. Supl. Table 7. GO for down regulated genes. Supl. Fig. 1. FPKM values for the two CCDS SMARCB1 transcripts for AT/RT and Controls. NCBI accession nomenclature is used. Supl. Fig. 2. RNA reads alignment at exon 2 of SMARCB1. Genomic region Chr. 22: 24134055- 24134081 is indicated between the two red vertical lines. The yellow region on the top, represents exon 2, with introns on both sides, shown in blue. Aligned paired reads are shown in blue, while single reads are shown in red or green. Dotted lines connect each end of a read, that have been mapped across exon-exon boundaries. [file 862039.f1.zip › 862039 Supl_Fig 2.pdf]

# SMARCB1

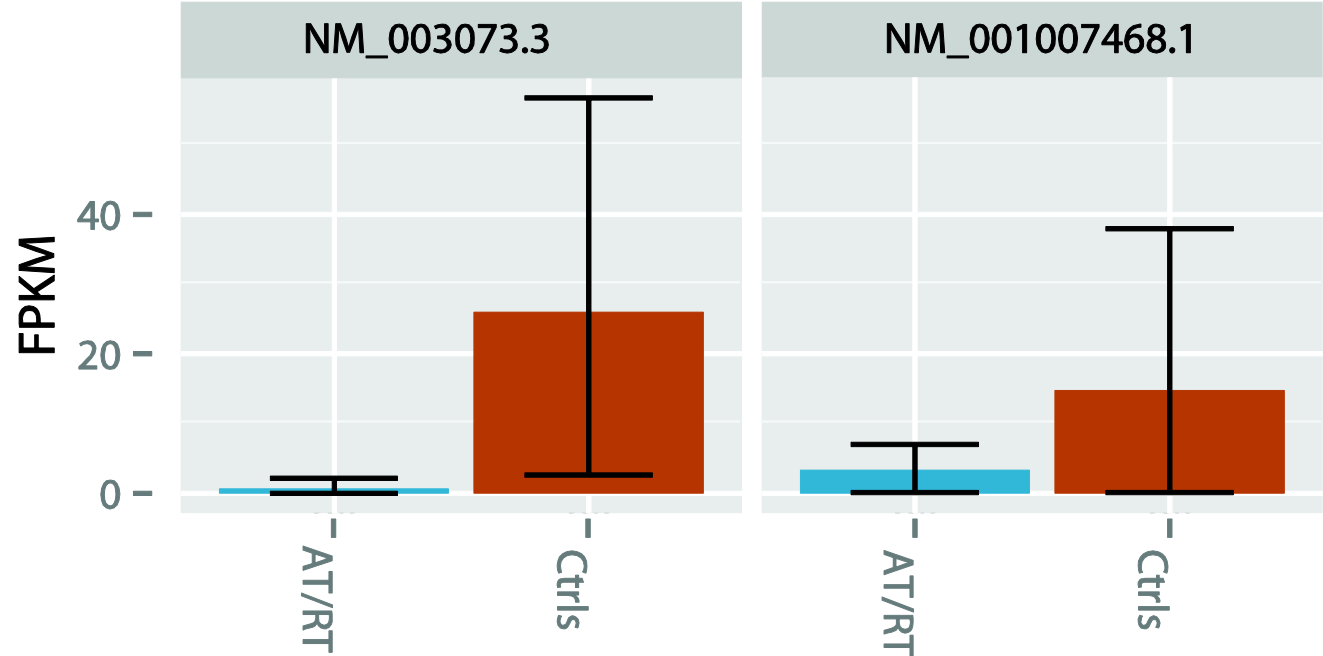

Supplement: Supplementary file 1 — Supl. Table 1A. Read mapping and coverage data. Exome-seq Data. Supl. Table 1B. Read mapping mRNA-seq data. Supl. Table 2. Germline variants with RNA variants and DE genes indicated. Supl. Table 3. Cuffdiff results, significantly DE genes with BMP-genes indicated. Supl. Table 4. Gene set overlap for up regulated genes. Supl. Table 5. Gene set overlap for down regulated genes. Supl. Table 6. GO for up regulated genes. Supl. Table 7. GO for down regulated genes. Supl. Fig. 1. FPKM values for the two CCDS SMARCB1 transcripts for AT/RT and Controls. NCBI accession nomenclature is used. Supl. Fig. 2. RNA reads alignment at exon 2 of SMARCB1. Genomic region Chr. 22: 24134055- 24134081 is indicated between the two red vertical lines. The yellow region on the top, represents exon 2, with introns on both sides, shown in blue. Aligned paired reads are shown in blue, while single reads are shown in red or green. Dotted lines connect each end of a read, that have been mapped across exon-exon boundaries. [file 862039.f1.zip › Supl_Fig 1.pdf]
